# Novel Antidiabetic Drugs and the Risk of Diabetic Retinopathy: A Systematic Review and Meta-Analysis of Randomized Controlled Trials

**DOI:** 10.3390/jcm13061797

**Published:** 2024-03-20

**Authors:** Artur Małyszczak, Joanna Przeździecka-Dołyk, Urszula Szydełko-Paśko, Marta Misiuk-Hojło

**Affiliations:** 1 Department and Clinic of Ophthalmology, Wroclaw Medical University, 50-367 Wroclaw, Poland; 2 Ophthalmology Clinical Center SPEKTRUM, Research and Development Center CREO, 53-334 Wroclaw, Poland; 3 Department of Optics and Photonics, Wroclaw University of Science and Technology, 50-370 Wroclaw, Poland

**Keywords:** diabetic retinopathy, GLP-1RA, DPP-4i, SGLT-2i, antidiabetic drugs

## Abstract

**Background**: The aim of this study is to compare the effect of sodium–glucose cotransporter-2 inhibitors (SGLT-2i), glucagon-like peptide-1 receptor agonists (GLP-1RA), and dipeptidyl peptidase-4 inhibitors (DPP-4i) on the risk of diabetic retinopathy (DR) in patients with type 2 diabetes (DM2). **Methods:** We systematically searched the databases Pubmed, Embase, and Clinicaltrials up to October 2, 2023, for randomized clinical trials (RCTs) of drugs from the GLP-1RA, SGLT-2i, and DPP-4i groups, with at least 24 weeks duration, including adult patients with DM2 and reported ocular complications. A pairwise meta-analysis was performed to calculate the odds ratio (OR) of DR incidents. **Results:** Our study included 61 RCTs with a total of 188,463 patients and 2773 DR events. Pairwise meta-analysis showed that included drug groups did not differ in the risk of DR events: GLP1-RA vs. placebo (OR 1.08; CI 95% 0.94, 1.23), DPP-4i vs. placebo (OR 1.10; CI 95% 0.84, 1.42), SGLT2i vs. placebo (OR 1.02; CI 95% 0.76, 1.37). Empagliflozin may be associated with a lower risk of DR, but this sub-analysis included only three RCTs (OR 0.38; 95% CI 0.17, 0.88, *p* = 0.02). **Conclusions:** Based on currently available knowledge, it is challenging to conclude that the new antidiabetic drugs significantly differ in their effect on DR complications.

## 1. Introduction

Diabetic retinopathy (DR) stands as one of the leading causes of visual impairment in developed countries [1]. Hyperglycemia plays an important role in the pathophysiology of DR as it affects vascular endothelial function [2]. In recent years, an increasing number of new antidiabetic drugs have become available. Besides their varying abilities to lower blood glucose levels, these drugs also exhibit diverse effects on the vascular endothelium, potentially influencing the onset and progression of DR [3,4,5]. The SUSTAIN 6 trial has indicated a higher incidence of DR complications with the use of Semaglutide compared to placebo [6]. However, some analyses do not support this relationship, suggesting the potential role of the rate of glucose-lowering as a contributing factor, as the magnitude of HbA1C reduction has been associated with increased DR risk in glucagon-like peptide-1 receptor agonists (GLP-1RA) treated population [7,8]. Previous meta-analyses of randomized clinical trials (RCTs) have suggested a potential association between the use of GLP-1RA and Canagliflozin and a higher risk of vitreous hemorrhage in patients with type 2 diabetes mellitus (DM2) [9,10]. Nonetheless, conflicting results from other studies challenge these findings [11,12,13,14]. The current body of evidence remains inconclusive. Considering the expected increase in the incidence of diabetes and its complications in the coming years, it is crucial to determine how new antidiabetic drugs may impact the risk of DR [15]. We conducted a pairwise meta-analysis and meta-regression of randomized clinical trials, including patients with DM2, comparing the risk of DR complications between new antidiabetic drugs sodium–glucose cotransporter-2 inhibitors (SGLT-2i), GLP-1RA, dipeptidyl peptidase-4 inhibitors (DPP-4i), and placebo. The aim of our study was to determine the potential impact of these drugs on the risk of DR complications. The secondary aim was to investigate whether other factors, such as differences in changes of glycated hemoglobin blood concentration (HbA1c) between intervention and control groups, HbA1C at baseline, body mass index (BMI) at baseline, age, and duration of diabetes, might contribute to variations in this risk.

## 2. Materials and Methods

We conducted our meta-analysis in accordance with the Preferred Reporting Items for Systematic Reviews and Meta-Analyses 2020 (PRISMA) [16]. The protocol of the systematic review was registered in the International Prospective Register of Systematic Reviews (PROSPERO) under the registration number CRD42022336459.

### 2.1. Search Strategy and Study Selection

We systematically searched the databases Pubmed, Embase, and Clinicaltrials.gov using the search strategy included in Text S1. Studies published up to 2 October 2023, were included. Only trials reported in English were included in our study. Two independent reviewers assessed titles, abstracts, and full texts using the Rayyan online tool [17]. Additionally, we examined the bibliographies of included papers. Inclusion criteria were as follows: a randomized clinical trial of at least 24 weeks duration, adult patients with DM2 reporting ocular complications, drugs from the SGLT-2i, GLP-1RA, and DPP-4i groups (Canagliflozin, Empagliflozin, Ertugliflozin, Dapagliflozin, Sotagliflozin, Luseogliflozin, Linagliptin, Saxagliptin, Teneligliptin, Alogliptin, Omarigliptin, Vildagliptin, Albiglutide, Lixisenatide, Semaglutide, Dulaglutide, Liraglutide, Efpeglenatide, Exenatide). A list of counted DR complications is available in Text S2.

### 2.2. Data Collection and Risk of Bias Assessment

Two independent reviewers collected data and assessed the risk of bias. In cases of conflicting opinions, a third reviewer resolved the conflict. Data were collected from full articles, protocols, clinical study reports, and ClinicalTrials.gov database. We collected the following data: author, publication year, trial name, intervention, control, mean age, percentage of male participants, number of subjects, follow-up duration, background treatment, characteristics of the patient population, HbA1C levels at baseline and their changes at the study endpoint for each group, BMI, and DR events. For the HbA1C endpoint, we selected the longest time point measurement where at least half of the study’s initial population remained. In the case of missing data, which only occurred for the variables analyzed in the meta-regression, the study was omitted from the calculation. The risk of bias was assessed using the Cochrane risk-of-bias tool for randomized trials (RoB 2) [18]. Five domains were analyzed: risk of bias arising from the randomization process, risk of bias due to deviations from the intended interventions (effect of assignment to intervention), missing outcome data, risk of bias in the measurement of the outcome, and risk of bias in the selection of the reported result.

### 2.3. Certainty Assessment

Certainty in the body of evidence for each outcome was assessed using the GRADE approach (The Grading of Recommendations Assessment, Development, and Evaluation) [19]. This method is used to rate the certainty of evidence in systematic reviews through the assessment of five domains: risk of bias, inconsistency, indirectness of evidence, imprecision of the effect estimates, and risk of publication bias. Evaluation of each domain can lower the level of evidence, as there are four levels: very low, low, moderate, and high.

### 2.4. Statistical Analysis

We conducted a pairwise meta-analysis using a random effects model to calculate the odds ratio (OR) and 95% confidence interval (95% CI) for the risk of diabetic retinopathy events between different drug groups and placebo. Sub-analyses were performed for drugs with three or more RCTs. The results of the meta-analysis were presented as a forest plot. To assess heterogeneity between studies, I^2^ statistics was used. Subgroup analyses and meta-regression were conducted to explore possible causes of heterogeneity. Sensitivity analysis was performed to determine the impact of individual studies on the OR of DR incidents. Publication bias was evaluated using funnel plot analysis and Egger regression. Meta-regression was performed to analyze the influence of factors HbA1C change during the trial between intervention and control, HbA1C at baseline, diabetes duration on baseline, BMI on baseline, age on baseline, and OR of DR incidents. Statistical analyses were conducted using Statistica v 13 (TIBCO Software Inc., Santa Clara, CA, USA) with plus kit v 5.0.96, under the license for Wroclaw Medical University.

## 3. Results

From the 13,694 preliminary studies found, we selected 966 studies for full-text analysis (Figure S1). Ultimately, 61 RCTs were included in the study (Table 1). A total of 188,463 subjects were included in the meta-analysis, with 2773 diabetic retinopathy events. The mean treatment duration was 1.57 years, and participants had an average diabetes duration of 9.91 years at baseline. On average, 58.4% of the subjects in each RCT were male. Characteristics of included studies are presented in Table 1, and HbA1C data is available in Table S1.

### 3.1. Risk of Bias

The majority of RCTs included in the study exhibited some concerns or a high risk of bias (50.8% vs. 37.7%, respectively). This was primarily attributed to the measurement methods for diabetic retinopathy complications, missing data, and the methods of analysis used to estimate the effect of assignment to intervention. The analyzed RCTs mostly did not perform regular fundoscopy, and some of them did not have pre-defined ocular complications. Most of the studies assessed in domain 2 (effect of assignment to intervention) showed some concerns due to uncertainty about the validity of the method of adverse events analysis. A significant portion of RCTs analyzed adverse events using an as-treated approach. Additionally, six studies had a high risk of bias due to an open-label design. Finally, only seven studies were assessed as low risk. RoB individual study ratings are available in Table S2.

### 3.2. Certainty Assessment

Assessments of certainty are presented in Table S3. Five out of twelve outcomes were graded as moderate certainty, and the rest were graded as low or very low. Indirectness was rated as serious in every outcome because most of the included RCTs differed in terms of background therapy and ophthalmic events, which were collected from adverse event summaries. Imprecision was assessed based on the absolute effect, as the analyzed trials had large populations and low event rates.

### 3.3. Pairwise Meta-Analysis

**Figure 1:**
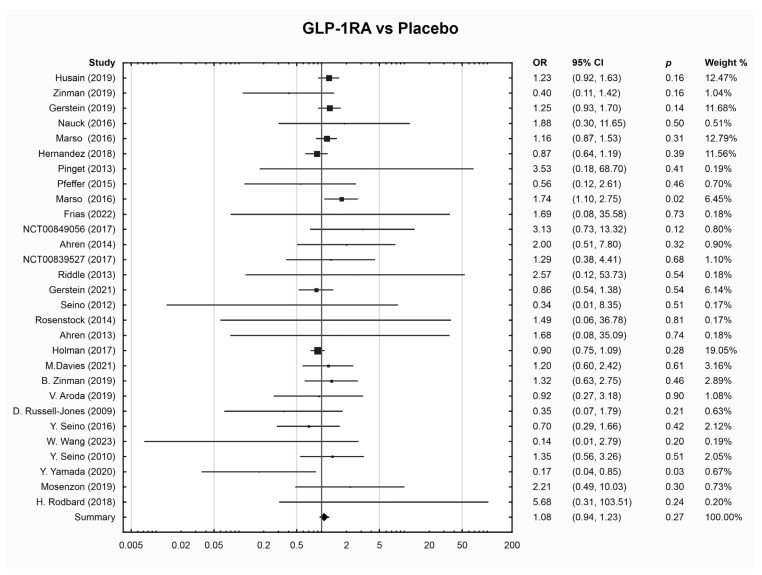
Pairwise meta-analysis, GLP-1RA vs. placebo [6,20,22,23,24,25,26,27,30,46,47,49,51,52,55,57,58,61,62,63,65,66,67,69,71,73,74].

**Figure 2:**
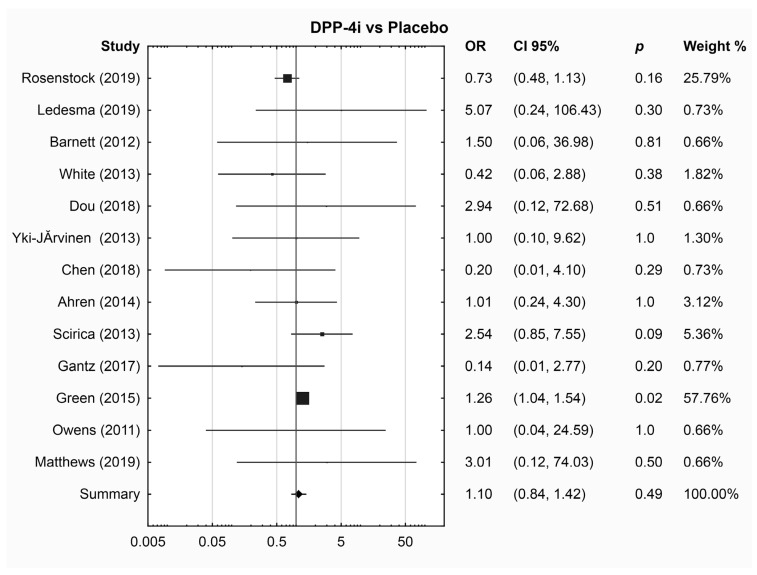
Pairwise meta-analysis, DPP-4i vs. placebo [37,39,40,41,43,44,45,47,50,53,54,56,59].

**Figure 3:**
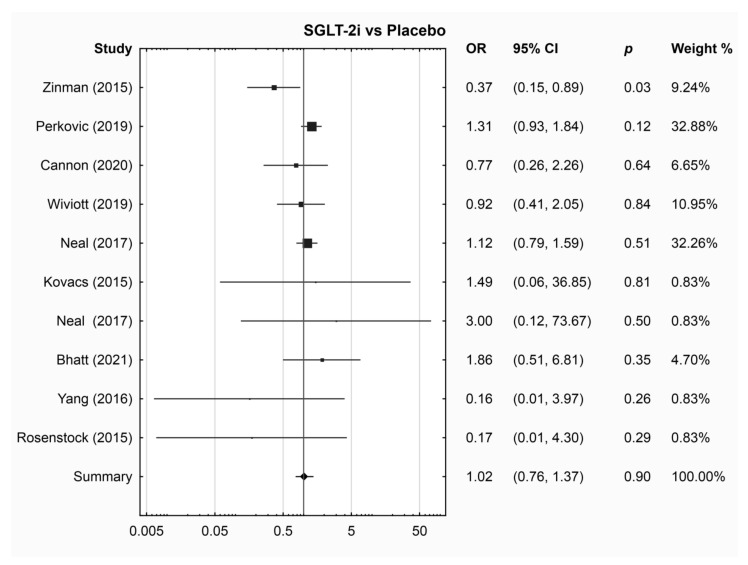
Pairwise meta-analysis, SGLT-2i vs. placebo [33,34,35,36,38,42,48,68,70].

The results of the meta-analysis results did not reveal a significant difference in the risk of DR events between any drug group and placebo (Table 2 and Figure 1, Figure 2, Figure 3 and Figure S2). Sub-analysis was performed for drugs with three or more RCTs (Figures S3–S10). Empagliflozin was associated with a lower risk of DR compared to placebo (OR 0.38; 95% CI 0.17, 0.88, *p* = 0.02); however, this sub-analysis included only three RCTs. SGLT-2i was not compared to GLP-1RA or DPP-4i due to the limited number of studies available for each comparison (one and two studies, respectively).

### 3.4. Heterogeneity Analysis

Strong heterogeneity was identified when comparing GLP-1RA to DPP-4i (I^2^ = 76.16%, *p* < 0.00). This is mainly due to the inclusion of the NCT01098539 study, which has an older population and a longer duration of diabetes compared to the other studies in this group (mean age at baseline 63.3 years vs. 55.6 years, mean duration at baseline 11.23 years vs. 7.3 years). The meta-regression results described below indicate that higher values of these two factors are associated with a lower risk of DR incidents with GLP-1RA when compared to DPP-4i. This relationship remains significant even after removing the NCT01098539 study from the analysis. While heterogeneity was also elevated in the Semaglutide vs. placebo comparison, it did not reach statistical significance (I^2^ = 37.88%, *p* = 0.12).

### 3.5. Publication Bias

Based on Egger’s test and visual inspection of the funnel plot, significant publication bias was found in Albiglutide vs. placebo (Egger *p* = 0.02), Linagliptin vs. placebo (Egger *p* = 0.24), and Liraglutide vs. placebo (Egger *p* = 0.28) comparisons. However, it is worth mentioning that these are analyses with a small number of studies (each less than 10 RCTs).

### 3.6. Sensitivity Analysis

In the sensitivity analysis, we assessed whether the inclusion of individual studies would result in a change in OR of DR incidents. When comparing DPP-4i with placebo, the exclusion of the CARMELINA trial would lead to a higher risk of DR incidents with DPP-4i use compared to placebo (OR 1.26; CI 95% 1.05, 1.53; *p* = 0.02). Additionally, when comparing Semaglutide vs. placebo, removal of the PIONEER 9 study would have resulted in a statistically significant increased risk of DR complications with Semaglutide, compared with placebo (OR 1.30; CI 95% 1.05, 1.60; *p* = 0.01). For the sub-analyses of the SGLT2 group, empagliflozin vs. placebo, sensitivity analysis also identified studies whose removal would significantly alter the outcomes. However, these are groups with a small number of studies (three).

### 3.7. Regression Analysis

A multivariate and univariate meta-regression of 44 RCTs found that there was no effect of the difference in HbA1C change between intervention and placebo, HbA1C on baseline, diabetes duration, age, or BMI on the risk of DR complications (Table S4). In univariate sub-analysis, a higher BMI at baseline was associated with an increased risk of DR complications in GLP-1RA use (Table S5). Additionally, a smaller difference in HbA1C change between DPP-4i and placebo use was linked to a higher risk of DR complications in DPP-4i use (Table S6). SGLT-2 inhibitors showed higher DR risk with higher HbA1C level at the start of the therapy (Table S7), and when comparing GLP-1RA to DPP-4i, older age and longer duration of diabetes at baseline lowered the DR risk in favor of the GLP-1RA group (Tables S8 and S9). Studies with missing data were excluded. Other sub-analyses were not included as they did not show a significant effect of the analyzed variables on the risk of DR incidents.

## 4. Discussion

Data from 61 RCTs involving a total of 188,463 patients and 2773 DR incidents were analyzed in our study. The analysis did not reveal an increased risk of DR events with the use of any drug group. The use of empagliflozin showed a potential association with a lowered risk of DR, but this finding is based on a sub-analysis involving only three RCTs. Further research with a larger number of studies in this subgroup may alter this observation.

### 4.1. SGLT-2i

The cardiovascular effects of SGLT-2i have garnered substantial attention, particularly through clinical trials like EMPA-REG OUTCOME, CANVAS, and DECLARE-TIMI 58, which demonstrated reductions in cardiovascular death and hospitalization for heart failure during SGLT-2i use [33,36,38]. These benefits are thought to be associated with their diuretic and natriuretic effects [75]. Additionally, SGLT-2i appears to exert protective effects on vascular endothelial function, potentially benefiting retinal health by enhancing glycemic control, managing hypertension and hyperlipidemia, and protecting the blood–retinal barrier and retinal capillaries [76]. Indeed, rodent studies have shown the beneficial effects of SGLT-2i on ophthalmic complications of diabetes, and human studies indicated the ability to reduce diabetic macular edema [77,78,79,80]. However, in our study, we did not observe a lower risk of DR complications with SGLT-2i use. These findings align with the meta-analysis and systematic review by Li et al., which also found no evidence of SGLT-2i providing benefits in reducing DR incidents or total ocular events in patients with type 2 diabetes [11]. The result of the meta-analysis by Zhou et al. partially supports this observation; compared to other antidiabetic drugs or placebo, the use of SGLT-2i was not associated with a reduction in the overall number of ocular complications in DM2 patients. However, subgroup analysis suggested that Ertugliflozin and Empagliflozin may reduce the risk of retinal disease and DR, accordingly. Canagliflozin, on the other hand, may increase the risk of vitreous disease compared to placebo [9]. Our study results are in agreement with the beneficial effect of Empagliflozin use, as the risk of DR complications was lower when compared to placebo. Unlike the aforementioned study published by Zhou et al., DR events were analyzed together and were not grouped, so the lack of effect of Canagliflozin on DR complications remains consistent with the results. It is important to note that our study included only one RCT on Ertugliflozin, so we did not perform a sub-analysis for this drug.

### 4.2. GLP-1RA

The effect of GLP-1RA extends beyond glycemic control alone, as GLP-1 receptors are present in many tissues, including the brain and heart [81]. Studies have also shown a protective effect on the retina by accelerating its regeneration and inhibiting the progression of DR [82,83]. Puddu et al. and Dorecka et al. detected GLP-1 receptors on the retinal pigment epithelium, suggesting a potential mechanism for the positive effect on reducing DR complications [84,85]. Additionally, Zhou et al. and Fu et al. showed a protective effect of GLP-1RA on retinal ganglion cells under conditions of high glucose levels [86]. However, not all studies agree with the protective effect of GLP-1RA on the diabetic retina. Hebsgaard et al. showed that GLP-1R expression is low in healthy eyes and virtually absent in eyes affected by proliferative diabetic retinopathy [87]. In our study, we did not show a higher risk of DR incidents with GLP-1 RA use compared to placebo. This result is consistent with previously performed meta-analyses of RCTs [12,13,14]. The exception was the study by Avgerinos et al., where the use of GLP-1RA was linked to a higher risk of vitreous hemorrhage [10]. The SUSTAIN 6 trial indicated a significantly higher rate of retinopathy complications in the Semaglutide group compared to placebo [6]. However, when analyzing the SUSTAIN 1–5 and Japanese trials, no significant difference was demonstrated when compared to the control groups. The authors of this analysis suggested that this phenomenon in the SUSTAIN 6 study might be attributed to a rapid reduction in HbA1C during the initial 16 weeks in patients treated with Semaglutide and insulin, particularly those already suffering from retinopathy with poor glycemic control [7]. In our study, we did not observe a higher risk of DR incidents associated with the use of Semaglutide compared to placebo.

### 4.3. DPP-4i

DPP-4i is suspected to have effects on the cardiovascular system and vascular endothelium [88]. Studies in rodents have indicated that DPP-4i may demonstrate retinoprotective effects [89,90]. Sitagliptin has been shown to have a beneficial effect on endothelial cell function during retinal inflammation [91]. For DPP-4i, their effect after topical administration in the form of eye drops is also being studied. Ramos et al. demonstrated the anti-oxidative and anti-inflammatory effects of topical administration of sitagliptin in diabetic retina [92]. However, some authors disagree on the protective effect of DPP-4i. Studies have suggested that prolonged use of DPP-4 inhibitors may induce vascular leakage, possibly by destabilizing barriers formed by retinal endothelial cells [93,94]. In a cohort study published in 2018, the use of DPP-4i did not result in a higher risk of DR incidents compared to other oral antidiabetic drugs at longer follow-up. Nonetheless, with a shorter duration of use (less than 12 months), the risk of DR complications was higher than in the never-use DPP-4i control group [95]. In a retrospective study, Chung et al. were among the first to show that the use of DPP-4i was an independent inhibitor of DR progression compared to the other antidiabetic drugs included in the study. However, this study included only eighty-two participants [96]. In another larger study, the authors demonstrated that DPP-4i did not increase the risk of DR progression compared to sulphonylureas [97]. In 2020, Taylor et al. published a meta-analysis of 18 studies, including RCTs, to determine the effect of DPP-4i on microvascular and macrovascular complications of diabetes. Among the data analyzed, there was no significant evidence of a protective effect of DPP-4i on the onset and progression of DR [98]. Consistent with these findings, our study also did not find an association between the use of DPP-4 inhibitors and the risk of DR incidents. In 2018, Tang et al. published a systematic review and meta-analysis of RCTs considering older and new antidiabetic drugs and their impact on DR complications in patients with DM2. Their pairwise meta-analysis indicated that the use of DPP-4i was associated with a higher risk of DR incidents compared to placebo. However, they suggested that this association was largely influenced by the inclusion of the TECOS trial and speculated that with the inclusion of more studies, this relationship might become statistically nonsignificant [99]. In our study, we included a larger number of RCTs and, as predicted by Tang et al., did not confirm this relationship. Our results are consistent concerning the other drug groups studied by the authors, where we also did not find any statistically significant difference in DR risk with the use of any drug group compared to placebo.

### 4.4. GLP-1RA vs. DPP-4i

We have found no association between GLP1-RA use and the risk of DR incidents when compared to DPP-4i. This result agrees with a cohort study that integrated data from Sweden and Denmark, which compared the risk of DR incidents in patients with a history of DR after their first prescription of GLP1-RA and DPP-4i. The authors found no association between the use of GLP-1RA and DR complications, with DPP-4i as an active comparator [100]. As there is no strong evidence of a higher risk of DR with either drug use, our result appears to be consistent with the available data.

### 4.5. Meta-Regression Analysis

In our study, the effect of the difference in HbA1C change between intervention and placebo on the risk of DR incidents was only demonstrated with DPP-4i use, where a greater reduction of HbA1C in the intervention group was associated with a lower risk of DR incidents. Previously, the opposite-worsening of DR was related to greater efficacy in lowering HbA1c by GLP-1RA [14]. HbA1C concentration was also significant in SGLT-2i vs. placebo comparison, where higher HbA1C on baseline resulted in higher DR risk in SGLT-2i use. This mechanism highlights the impact of high glycemia on ocular complications. In addition, we have demonstrated that there was also a higher DR risk during GLP-1RA use in patients with higher BMI. The association of BMI with DR complications has been previously described; however, results remain controversial [101,102,103]. GLP-1RA and DPP-4i comparison showed strong heterogeneity. It may be explained by our meta-regression results, which showed that older age and longer duration of diabetes on baseline were related to lower DR risk in the GLP-RA group and higher in DPP-4i. The lack of significant correlations when considering all included RCTs may be attributed to the different mechanisms of action of drug groups or the limited number of studies analyzed.

### 4.6. Strengths and Limitations

Strengths: The study includes a significant number of 61 RCTs and a large population of 188,463 subjects. In addition, the meta-regression performed allowed us to determine the effect of additional factors on the risk of DR with the use of the studied drug groups. In the subgroup analysis, we determined the risk of DR when using specific drugs, not only whole groups. Limitations: A notable limitation of our study is that the majority of included trials were primarily designed to evaluate the impact of tested drugs on cardiovascular events or glycemic control. Detailed fundoscopic examinations were conducted in only 19 of the included studies. DR endpoints came primarily from adverse events reporting, which may cause the number of DR events to be significantly underreported. Most of the studies did not report data on pre-existing retinopathy, so we could not explore tested drug effects in this population. As we included less than three studies, each comparing SGLT-2i with DPP-4i and GLP-1RA, we were not able to compare these drug groups.

## 5. Conclusions

In light of currently available knowledge, it is challenging to conclude that the new antidiabetic drugs differ significantly in their effect on diabetic retinopathy complications. The available data suggest a potential decrease in the risk of diabetic retinopathy incidents with empagliflozin use, but more studies are needed to confirm this observation. Controlling glycemia may offer potential benefits in reducing this risk when using incretin-based drugs and SGLT-2i, and using GLP-1RA in older populations may be beneficial compared to using DPP-4i. Studies show potential mechanisms by which these drugs could protect the retina, but most of the available randomized trials do not support these statements and do not include a detailed ophthalmic evaluation. Further RCTs, including detailed ophthalmic evaluation, are required to assess the impact of new antidiabetic drugs on diabetic retinopathy accurately. This is particularly important in light of their increasing use and the growing number of people suffering from diabetes.

## Figures and Tables

**Table 1:** Characteristics of included studies.

First Author	Year	CTID	Name	Number of Patients	Time in Trial (Years)	Intervention	Comparator	Characteristics of Participants	Background Treatment	Male (%)	Mean Age (Years)	Mean Diabetes Duration (Years)	Mean BMI	Mean HbA1C (%)	IG DR Incidents/Group Size	CG DR Incidents/Group Size
M. Husain [20]	2019	NCT02692716	PIONEER 6	3182	1.6	Semaglutide	Placebo	Adults with DM2 at high cardiovascular risk	Standard-of-care treatment	68.4	66.0	14.90	32.30	8.20	109/1591	90/1592
J. Rosenstock [21]	2019	NCT02607865	PIONEER 3	1861	1.5	Semaglutide	Sitagliptin	Adults with DM2 taking a stable dosage of metformin with or without sulfonylurea	Metformin ± sulfonylurea	52.8	58.0	8.60	32.50	8.30	83/1395	36/466
B. Zinman [22]	2019	NCT03086330	SUSTAIN 9	301	0.6	Semaglutide	Placebo	Adults with DM2 inadequately controlled despite at least 90 days of treatment with an SGLT-2 inhibitor	Standard-of-care treatment, including SGLT-2 inhibitor treatment	58.3	57.0	9.70	31.90	8.00	3/150	8/151
H. Gerstein [23]	2019	NCT01394952	REWIND	9892	5.4	Dulaglutide	Placebo	Adults with DM2 with a previous CV event, evidence of CV disease, or > 2 CV risk factors	Standard-of-care treatment	53.7	66.0	10.00	32.30	7.30	95/4949	76/4952
M. Nauck [24]	2016	NCT00849017	HARMONY-2	301	3	Albiglutide	Placebo	Adults with DM2 inadequately controlled by diet and exercise	none	55.1	52.9	3.96	33.53	8.00	5/200	1/101
S. Marso [25]	2016	NCT01179048	LEADER	9336	3.8	Liraglutide	Placebo	Adults with DM2 at high cardiovascular risk	Standard-of-care treatment	64.3	64.3	12.80	32.50	8.70	106/4668	92/4672
A. Hernandez [26]	2018	NCT02465515	HARMONY	9432	1.6	Albiglutide	Placebo	Adults with DM2 with cardiovascular disease	Standard-of-care treatment	69.4	64.1	14.15	32.30	8.70	78/4717	89/4715
M. Pinget [27]	2013	NCT00763815	GETGOAL-P	484	1.6	Lixisenatide	Placebo	Adults with DM2 who were treated with pioglitazone	Pioglitazone ± metformin	52.5	55.8	8.10	33.92	8.07	3/323	0/161
L. Ji [28]	2021	NCT03061214	SUSTAIN CHINA	867	0.6	Semaglutide	Sitagliptin	Adults with DM2 treated with metformin monotherapy	Metformin	57	53	6.36	28.2	8.1	36/577	10/290
R. Pratley [29]	2010	NCT00700817	LIRA-DPP-4	658	1.0	Liraglutide	Sitagliptin	Adults with DM2 previously treated with metformin monotherapy	Metformin	52.9	55.3	6.2	32.8	8.4	7/439	1/219
M. Pfeffer [30]	2015	NCT01147250	ELIXA	6063	1.8	Lixisenatide	Placebo	Adults with DM2 who had had a myocardial infarction or who had been hospitalized for unstable angina within the previous 180 days	Standard-of-care treatment	69.3	60.3	9.29	30.16	7.68	2/3031	4/3032
C. Son [31]	2021		CANTABILE	162	0.5	Teneligliptin	Canagliflozin	Adults with DM2 and one or more metabolic risk factors	Standard-of-care treatment	67.55	56	6.3	29	7.8	2/80	0/82
H. Rodbard [32]	2019	NCT02863328	PIONEER 2	819	1.0	Semaglutide	Empagliflozin	Adults with DM2 receiving a stable dose of metformin	Metformin	50.5	58	7.4	32.8	8.1	14/410	5/409
B. Zinman [33]	2015	NCT01131676	EMPA-REG OUTCOME	7020	3.1	Empagliflozin	Placebo	Adults with DM2, established ASCVD, and estimated glomerular filtration rate ≥30 mL/min/1.73 m^2^	Standard-of-care treatment	71.5	63.1	82% >5 y	30.625	8.0775	8/4687	11/2333
V. Perkovic [34]	2019	NCT02065791	CREDENCE	4397	2.6	Canagliflozin	Placebo	Adults with DM2 with chronic kidney disease	Standard-of-care treatment	66.1	63	15.8	31.3	8.3	78/2200	60/2197
C. Cannon [35]	2020	NCT01986881	VERTIS CV	8238	3.5	Ertugliflozin	Placebo	Adults with DM2, established ASCVD	Standard-of-care treatment	70	64.4	13	31.95	8.2	8/5493	5/2745
S. Wiviott [36]	2019	NCT01730534	DECLARE-TIMI58	17,143	4.2	Dapagliflozin	Placebo	Adults with DM2, creatinine clearance of 60 mL/min, ASCVD, or multiple risk factors for it	Standard-of-care treatment	62.6	63.9	10.5	32.05	8.3	11/8574	12/8569
J. Rosenstock [37]	2019	NCT01897532	CARMELINA	6979	2.2	Linagliptin	Placebo	Adults with DM2, high CV, and renal risk	Standard-of-care treatment	62.9	65.9	14.75	31.35	7.95	36/3494	49/3485
B. Neal [38]	2017	NCT01032629	CANVAS	4327	4.2	Canagliflozin	Placebo	Adults with DM2, history or high risk of CV disease	Standard-of-care treatment	66	62.4	16	33.1	8.3	106/2886	47/1441
G. Ledesma [39]	2019	NCT02240680		302	0.5	Linagliptin	Placebo	Adults with DM2 treated with basal insulin maintained at a stable dose for 4 weeks prior to randomization	Insulin. Optional metformin ± alpha-glucosidase inhibitor	60.6	72.4	76% >10 y	28	8.2	2/151	0/151
S. Marso [6]	2016	NCT01720446	SUSTAIN 6	3297	2.0	Semaglutide	Placebo	Adults with DM2, established CVD, chronic heart failure, or chronic kidney disease	Standard-of-care treatment	60.7	64.6	13.9	32.8	8.7	50/1648	29/1649
A. Barnett [40]	2012	NCT00757588		455	0.5	Saxagliptin	Placebo	Adults with DM2 inadequately controlled on a stable dose of insulin	Insulin ± metformin	41.3	58	12	32.2	8.65	1/304	0/151
W. White [41]	2013	NCT00968708	EXAMINE	5380	1.5	Alogliptin	Placebo	Adults with DM2 had an acute coronary syndrome within 15 to 90 days before randomization	Standard-of-care treatment	67.9	61	7.2	28.7	8	1/2701	3/2679
C. Kovacs [42]	2015	NCT01210001	EMPA-REG EXTEND PIO	498	0.5	Empagliflozin	Placebo	Adults with DM2 inadequately controlled on a diet and exercise regimen, receiving pioglitazone monotherapy	Pioglitazone ± metformin	48.4	54.5	87% >1 y	29.2	8.09	1/333	0/165
J. Dou [43]	2018	NCT02273050	START	425	0.5	Saxagliptin	Placebo	Adults with DM2 inadequately controlled with diet and exercise	none	64.3	50.25	0.845	26.6	9.45	1/215	0/210
H. Yki-Järvinen [44]	2013	NCT00954447		1261	1.0	Linagliptin	Placebo	Adults with DM2 inadequately controlled on treatment with basal insulin	Basal insulin, standard-of-care treatment	52.2	60	86% >5 y	31	8.3	1/631	1/630
Y. Chen [45]	2018	NCT02104804	SUPER	465	0.5	Saxagliptin	Placebo	Adults with DM2 inadequately controlled with a stable regimen of insulin or insulin plus metformin	Insulin ± metformin	45.2	59.1	13.4	26.2	8.53	0/234	2/231
J. Frias [46]	2022	NCT03353350	AMPLITUDE-M	406	1.1	Efpeglenatide	placebo	Adults with DM2, inadequately controlled with diet and exercise	none	53.9	58.5	5.1	34.2	8.05	2/304	0/102
*	2017	NCT00849056		301	3.0	Albiglutide	Placebo	Adults with DM2	Pioglitazone ± metformin	59.8	55	7.961	34.12	8.11	7/150	2/151
*	2017	NCT01098539		495	1.0	Albiglutide	Sitagliptin	Adults with DM2, renally impaired and inadequately controlled with diet and exercise or their antidiabetic therapy	Metformin, sulfonylurea, or thiazolidinediones	53.7	63.3	11.23	30.39	8.18	12/249	50/246
B. Neal [38]	2017	NCT01989754	CANVAS-R	5807	1.8	Canagliflozin	Placebo	Adults with DM2, history or high risk of CVD	Standard-of-care treatment	62.8	64	13.7	31.9	8.3	1/2904	0/2903
B. Ahren [47]	2014	NCT00838903	HARMONY 3	705	2.0	Albiglutide, Sitagliptin	Placebo	Adults with DM2 inadequately controlled with background metformin	Metformin	47.6	54.5	6.125	32.6	8.125	14/302 ^a^, 7/302 ^s^, 2/101 ^p^	
*	2014	NCT00839527		386	3.0	Albiglutide	Placebo	Adults with DM2	Glimepiride + metformin	53.2	55.2	9	32.5	8.2	10/271	3/115
D. Bhatt [48]	2021	NCT03315143	SCORED	10,577	1.3	Sotagliflozin	Placebo	Adults with DM2, chronic kidney disease, and additional CVD risk factors	Standard-of-care treatment	55.1	69	N/a	31.8	8.3	6/5291	3/5286
M. Riddle [49]	2013	NCT00715624	GETGOAL-L	495	0.5	Lixisenatide	Placebo	Adults with DM2 inadequately controlled with basal insulin with or without metformin	Insulin ± Metformin	46.1	57	12.5	32.1	8.4	2/328	0/167
B. Scirica [50]	2013	NCT01107886	SAVOR- TIMI 53	16,492	2.1	Saxagliptin	Placebo	Adults with DM2, a history of established CVD, or multiple risk factors for vascular disease	Standard-of-care treatment	66.9	65	10.3	31	8	11/8280	4/8212
H. Gerstein [51]	2021	NCT03496298	AMPLITUDE-O	4073	1.8	Efpeglenatide	Placebo	Adults with DM2 and either a history of CVD or current kidney disease plus at least one other cardiovascular risk factor	Standard-of-care treatment	67	64.5	15.4	32.7	8.91	47/2717	27/1359
Y. Seino [52]	2012	NCT00866658	GETGOAL-L-ASIA	311	0.5	Lixisenatide	Placebo	Adults with DM2 currently on stable basal insulin therapy with or without a sulfonylurea	Insulin ± sulfonylureas	47.9	58.4	13.92	25.26	8.53	0/154	1/157
I. Gantz [53]	2017	NCT01703208		4192	1.8	Omarigliptin	Placebo	Adults with DM2, established CVD	Standard-of-care treatment	70.2	63.6	12.05	31.3	8.01	0/2092	3/2100
J. Green [54]	2015	NCT00790205	TECOS	14,540	3.0	Sitagliptin	Placebo	Adults with DM2 with established CVD, treated with stable doses of one or two oral antihyperglycemic agents	One or two oral antihyperglycemic agents (metformin, pioglitazone, or sulfonylurea) or insulin	70.7	65.5	11.6	30.2	7.2	226/7332	180/7339
J. Rosenstock [55]	2014	NCT00713830	GETGOAL-S	859	0.5	Lixisenatide	Placebo	Adults with DM2 currently receiving an SU with or without metformin	Sulfonylurea ± Metformin	50.5	57.2	9.45	30.25	8.25	1/574	0/285
D. Owens [56]	2011	NCT00602472		1055	0.5	Linagliptin	Placebo	Adults with DM2 inadequately controlled by metformin and sulphonylurea combination treatment	Sulfonylurea + Metformin	47.2	58.1	73% >5 y	28.33	8.14	1/792	0/263
B. Ahren [57]	2013	NCT00712673	GETGOAL-M	680	0.5	Lixisenatide	Placebo	Adults with DM2 inadequately controlled on metformin with a dose of at least 1.5 g/day for at least 3 months	Metformin	43.1	57.4	6.11	32.91	8.06	2/510	0/170
R. Holman [58]	2017	NCT01144338	EXSCEL	14,716	3.2	Exenatide	Placebo	Adults with DM2	Standard-of-care treatment	62	61.9	12	31.75	8	214/7344	238/7389
D. Matthews [59]	2019	NCT01528254	VERIFY	1999	5.0	Vildagliptin	Placebo	Adults with DM2	Metformin	47	54.3	0.28	31.1	6.7	1/998	0/1001
M. Sugawara [60]	2023		J-SELECT	599	1	Luseogliflozin	DPP-4i	Adults with DM2	Standard-of-care treatment	65.4	57.75	4.45		7.65	1/300	0/299
M. Davies [61]	2021	NCT03552757	STEP 2	1210	1.3	Semaglutide	Placebo	Adults with DM2	none	49.1	55	8	35.7	8.1	27/805	11/402
B. Zinman [62]	2019	NCT03021187	PIONEER 8	731	1	Semaglutide	Placebo	Adults with DM2 inadequately controlled with insulin ± metformin	Insulin ± metformin	54	61	15	31	8.2	36/546	9/184
V. Aroda [63]	2019	NCT02906930	PIONEER 1	703	0.5	Semaglutide	Placebo	Adults with DM2 managed only by diet and exercise	none	50.8	55	3.5	31.8	8	9/525	3/178
Y. Seino [64]	2018	NCT02254291		308	0.58	Semaglutide	Sitagliptin	Adults with DM2 treated with diet and exercise only or oral antidiabetic drug monotherapy	none	76.3	58.3	8	25.4	8.1	6/205	4/103
D. Russell-Jones [65]	2009	NCT00331851	LEAD-5 met+SU	581	0.5	Liraglutide	Placebo	Adults with DM2 treated with oral glucose-lowering drugs for at least 3 months before screening	Metformin + Glimepiryde	53	57.5	9.3	30.85	8.3	2/230	3/114
Y. Seino [66]	2016	NCT01572740		257	0.7	Liraglutide	Placebo	Adults with DM2 on stable insulin therapy in addition to diet and exercise	Insulin	56	60.5	14.5	25.6	8.8	9/127	13/130
W. Wang [67]	2023	NCT04591626	AWARD-CHN3	291	0.5	Dulaglutide	Placebo	Adults with DM2 inadequately controlled with a stable dose of basal insulin glargine once daily with metformin ± or acarbose	Insulin, metformin, and/or acarbose	62.5	58.1	11.8	25.9	8.6	0/144	3/147
*	2023	NCT04017832	PIONEER 12	1441	0.5	Semaglutide	Sitagliptin	Adults with DM2 inadequately controlled on metformin ≥ 60 days prior to the day of screening	Metformin	58.3	53.3	n/a	n/a	n/a	0/1080	1/358
W. Yang [68]	2016	NCT01095666		444	0.5	Dapagliflozin	Placebo	Adults with DM2 inadequately controlled on metformin	Metformin	54.3	53.8	4.9	26.1	8.13	0/299	1/145
Y. Seino [69]	2011	NCT00395746		264	1	Liraglutide	Placebo	Adults with DM2 inadequately controlled on diet therapy and one SU agent	Sulfonylurea	64	59.7	10.3	24.9	8.82	19/176	7/88
J. Rosenstock [70]	2015	NCT01011868	EMPA-REG BASAL	494	1.5	Empagliflozin	Placebo	Adults with DM2 inadequately controlled despite treatment with basal glargine or detemir insulin ± metformin and/or sulphonylurea use	Basal insulin, with or without metformin ± sulphonylureas	56	58.8	89% >5 y	32.2	8.2	0/324	1/170
Y. Yamada [71]	2020	NCT03018028	PIONEER 9	243	1	Semaglutide	Placebo	Adults with DM2	none	79	59	7.6	25.9	8.2	2/146	4/49
T. Pieber [72]	2019	NCT02849080	PIONEER 7	504	1	Semaglutide	Sitagliptin	Adults with DM2 receiving stable daily doses of one or two glucose-lowering drugs	Standard-of-care treatment	57	57	8.8	31.5	8.3	6/253	6/250
O. Mosenzon [73]	2019	NCT02827708	PIONEER 5	324	0.5	Semaglutide	Placebo	Adults with DM2 with moderate renal impairment, receiving metformin or sulfonylurea, or both, or basal insulin with or without metformin	Metformin ± sulphonyloureas or insulin ± metformin	48	70	14	32.4	8	5/163	2/161
H. Rodbard [74]	2018	NCT02305381	SUSTAIN 5	397	0.6	Semaglutide	Placebo	Adults with DM2 inadequately controlled with basal insulin ± metformin	Insulin ± metformin	56.1	58.8	13.3	32.2	8.4	5/263	0/133

CTID, ClinicalTrials.gov identifier; DM2, type 2 diabetes; IG, Intervention group; CG, control group; DR, diabetic retinopathy; CV, cardiovascular; CVD, cardiovascular disease; ASCVD, Atherosclerotic Cardiovascular Disease; mL, milliliter; min, minute; y, year; ±, with or without; *, no publication has been found; ^a^, albiglutide group; ^s^, sitagliptin group; ^p^, placebo group.

**Table 2:** Pairwise meta-analysis summary.

Number of Trials	Intervention	Comparator	OR	95% CI	*p*	I^2^ Statistics	Egger’s Test *p*
29	GLP-1RA	Placebo	1.08	0.94; 1.23	0.27	13.82%	0.74
13	DPP-4i	Placebo	1.10	0.84; 1.42	0.49	7.84%	0.65
10	SGLT-2	Placebo	1.02	0.76; 1.37	0.9	19.34%	0.23
8	GLP-1RA	DPP-4i	0.85	0.44; 1.64	0.63	76.16%	0.79
3	Canagliflozin	Placebo	1.22	0.96; 1.56	0.11	0.00%	0.54
3	Empagliflozin	Placebo	0.38	0.17; 0.88	0.02	0.00%	0.82
4	Linagliptin	Placebo	0.77	0.51; 1.17	0.22	0.00%	0.24
4	Saxagliptin	Placebo	1.93	0.76; 4.91	0.17	0.00%	0.43
5	Albiglutide	Placebo	1.15	0.74; 1.79	0.54	15.45%	0.02
6	Lixisenatide	Placebo	0.99	0.35; 2.77	0.98	0.00%	0.21
9	Semaglutide	Placebo	1.18	0.84; 1.66	0.33	37.88%	0.48
4	Liraglutide	Placebo	1.06	0.78; 1.45	0.70	7.92%	0.29

OR, odds ratio; CI, confidence interval.

## Data Availability

Data sharing is not applicable.

## Supplementary Materials

The following supporting information can be downloaded at: https://www.mdpi.com/article/10.3390/jcm13061797/s1, Text S1: Search strategy; Text S2: Diabetic retinopathy related adverse events; Figure S1: Prisma flow diagram; Table S1: HbA1C on baseline and its change at the end of the study; Table S2: Risk of bias assessment; Table S3: Certainty assessment; Figure S2: Pairwise meta-analysis, GLP-1RA vs. DPP-4i [21,28,29,47,64,72]; Figure S3: Pairwise meta-analysis, Semaglutide vs. placebo [6,20,22,61,62,63,71,73,74]; Figure S4: Pairwise meta-analysis, Liraglutide vs. placebo [25,65,66,69]; Figure S5: Pairwise meta-analysis, Albiglutide vs. placebo [24,26,47]; Figure S6: Pairwise meta-analysis, Lixisenatide vs. placebo [27,30,49,52,55,57]; Figure S7: Pairwise meta-analysis, Linagliptin vs. placebo [37,39,44,56]; Figure S8: Pairwise meta-analysis, Saxagliptin vs. placebo [40,43,45,50]; Figure S9: Pairwise meta-analysis, Canagliflozin vs. placebo [34,38]; Figure S10: Pairwise meta-analysis, Empagliflozin vs. placebo [33,42,70]; Table S4: Meta-regression of prespecified trials characteristics on odds ratio of diabetic retinopathy incidents. Multivariate model; Table S5: Meta-regression of prespecified trials characteristics on odds ratio of diabetic retinopathy incidents. Univariate model. GLP-1RA vs. placebo; Table S6: Meta-regression of prespecified trials characteristics on odds ratio of diabetic retinopathy incidents. Univariate model. DPP-4i vs. placebo; Table S7: Meta-regression of prespecified trials characteristics on odds ratio of diabetic retinopathy incidents. Univariate model. SGLT-2i vs. placebo; Table S8: Meta-regression of prespecified trials characteristics on odds ratio of diabetic retinopathy incidents. Univariate model. GLP-1RA vs. DPP-4i; Table S9: Meta-regression of prespecified trials characteristics on odds ratio of diabetic retinopathy incidents. Univariate model. GLP-1RA vs. DPP-4i; Figure S11: Prisma checklist. Part 1 [16]; Figure S12: Prisma checklist. Part 2 [16].
