# Supplementary material for: Novel Antidiabetic Drugs and the Risk of Diabetic Retinopathy: A Systematic Review and Meta-Analysis of Randomized Controlled Trials
Source: J Clin Med. 2024 Mar 20;13(6):1797. doi: 10.3390/jcm13061797 (PMC10971133; doi:10.3390/jcm13061797)
Supplement: Supplementary file 1 [file jcm-13-01797-s001.zip › jcm-2906815-supplementary.pdf]

Text S1. Search strategy.

1.1 Pubmed

(Sodium-Glucose Transporter 2 Inhibitors[MeSH] OR SGLT-2 inhibitor* OR SGLT-2 OR SGLT2 OR sodium-glucose transporter 2 inhibitor* OR sodium-glucose transporter 2 antagonist* OR sodium glucose transporter ii inhibitor* OR Sodium Glucose co-transporter* OR Sodium dependent glucose cotransporter* OR sodium-glucose cotransporter* OR sodium-glucose transporter 2 antagonist* OR dapagliflozin OR canagliflozin OR empagliflozin OR remogliflozin OR ertugliflozin OR tofogliflozin OR Ipragliflozin OR Luseogliflozin OR Sotagliflozin) OR (Dipeptidyl-Peptidase IV Inhibitors[MeSH] OR Dipeptyl peptidase 4 inhibitor* OR Dipeptyl-peptidase IV inhibitor* OR DPP-4 inhibitor* OR DPP-4i OR DPP-IV OR DPP 4 OR DPP IV OR Gliptin* OR vildagliptin OR sitagliptin OR saxagliptin OR linagliptin OR alogliptin OR teneligliptin OR gemigliptin OR Anagliptin OR omarigliptin OR trelagliptin OR omarigliptin OR evogliptin) OR (Glucagon-Like Peptide-1 Receptor[MeSH] OR glucagon like peptide 1 receptor agonist* OR Glucagon-like peptide-1 receptor agonist* OR Glucagon-like peptide agonist* OR GLP-1 receptor agonist* OR GLP-1 agonist* OR GLP-1 OR GLP-1RA OR GLP-1 OR exenatide OR liraglutide OR lixisenatide OR semaglutide OR dulaglutide OR albiglutide OR Efpeglenatide) AND (Diabetes Mellitus, Type 2[MeSH] OR Type 2 diabetes OR non insulin dependent diabetes mellitus OR noninsulin dependent diabetes mellitus OR T2DM OR T2D OR NIDDM OR Diabetes type 2) AND (Randomized Controlled Trial[MeSH] OR Controlled clinical trial OR RCT OR random* OR Trial OR placebo) NOT (review OR meta-analysis OR meta analysis) NOT (animals[MeSH] NOT humans[MeSH])

1.2 Embase

('Sodium-Glucose Transporter 2 Inhibitor*' OR 'SGLT-2 inhibitor*' OR SGLT-2 OR SGLT2 OR SGLT-2* OR 'sodium-glucose transporter 2 inhibitor*' OR 'sodium-glucose transporter 2 antagonist*' OR 'sodium glucose transporter ii inhibitor*' OR 'Sodium Glucose co-transporter*' OR 'Sodium dependent glucose cotransporter*' OR 'sodium-glucose cotransporter*' OR 'sodium-glucose transporter 2 antagonist*' OR dapagliflozin OR canagliflozin OR empagliflozin OR remogliflozin OR ertugliflozin OR tofogliflozin OR Ipragliflozin OR Luseogliflozin OR Sotagliflozin) OR ('Dipeptidyl-Peptidase IV Inhibitor*' OR 'Dipeptyl peptidase 4 inhibitor*' OR 'Dipeptyl-peptidase IV inhibitor*' OR 'DPP-4 inhibitor' OR DPP-4i OR DPP-IV OR 'DPP 4' OR 'DPP IV' OR Gliptin* OR vildagliptin OR sitagliptin OR saxagliptin OR linagliptin OR alogliptin OR teneligliptin OR gemigliptin OR Anagliptin OR omarigliptin OR trelagliptin OR omarigliptin OR evogliptin) OR ('Glucagon-Like Peptide-1 Receptor*' OR 'glucagon like peptide 1 receptor agonist*' OR 'Glucagon-like peptide-1 receptor agonist*' OR 'Glucagon-like peptide agonist*' OR 'GLP-1 receptor agonist*' OR GLP-1 agonist* OR GLP-1 OR GLP-1RA OR GLP-1 OR exenatide OR liraglutide OR lixisenatide OR semaglutide OR dulaglutide OR albiglutide OR Efpeglenatide) AND ('Type 2 diabetes' OR 'non insulin dependent diabetes mellitus' OR 'noninsulin dependent diabetes mellitus' OR T2DM OR T2D OR NIDDM OR 'Diabetes type 2') AND (Randomized Controlled Trial OR Controlled clinical trial OR RCT OR random* OR Trial OR placebo) NOT (review OR meta-analysis OR meta analysis) NOT ('animal'/exp NOT 'human'/exp) AND ([Controlled Clinical Trial]/lim OR [Randomized Controlled Trial]/lim)

1.3 ClinicalTrials.gov

study types: interventional
study results: studies with results
age: Adult, Senior
conditions: type 2 diabetes
phase: 3, 4

Text S2. Diabetic retinopathy related adverse events.

- Diabetic Retinopathy
- Retinopathy proliferative
- Vitreous haemorrhage
- Retinal haemorrhage
- Diabetic retinal edema
- Macular edema
- Maculopathy
- Macular ischemia
- Cystoid macular edema
- Diabetic retinal edema
- Simple retinopathy
- Diabetic blindness
- Retinopathy (if diabetic retinopathy is not mentioned)
- Maculopathy
- Need for retinal photocoagulation
- Need for treatment with intravitreal agent
- Onset of diabetes- related blindness

Figure S1. Prisma flow diagram.

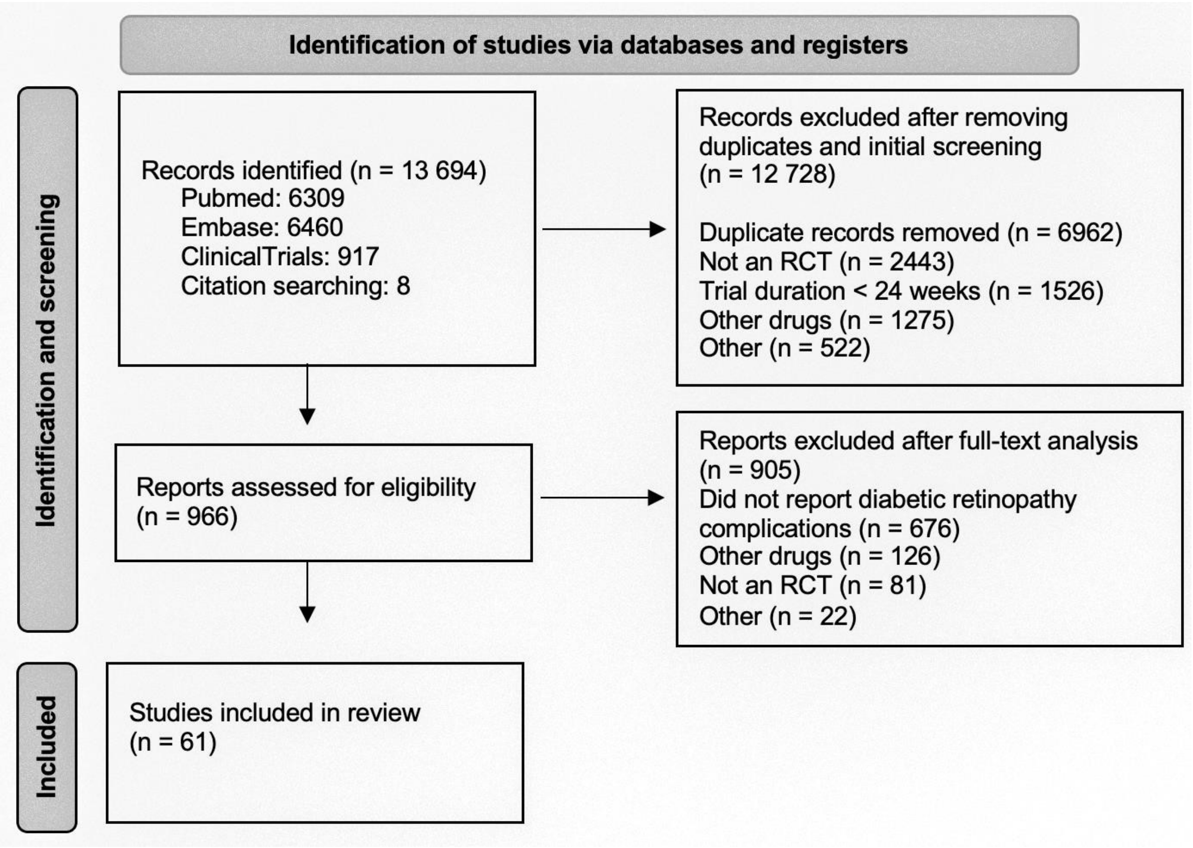

Table S1. HbA1C on baseline and its change at the end of the study.

First Author	CTID	Name	Intervention	Comparator	Overall Mean HbA1C at baseline	Mean HbA1C at baseline IG	HbA1C change IG	Mean HbA1C at baseline CG	HbA1C change CG	HbA1C change IG vs CG
M.Husain	NCT02692716	PIONEER 6	Semaglutide	placebo	8.20	8.20	-1.00	8.20	-0.30	-0.70
J.Rosenstock	NCT02607865	PIONEER 3	Semaglutide	Sitagliptin	8.30	8.33	-0.86	8.30	-0.70	-0.16
B. Zinman	NCT03086330	SUSTAIN 9	Semaglutide	placebo	8.00	8.00	-1.50	8.10	-0.10	-1.40
HC. Gerstein	NCT01394952	REWIND	Dulaglutide	placebo	7.30	7.30	-0.46	7.40	0.16	-0.62
	NCT00849017	HARMONY-2	Albiglutide	placebo	8.00	8.10	-0.81	8.00	0.18	-0.99
S. Marso	NCT01179048	LEADER	Liraglutide	placebo	8.70	8.70	n/a	8.70	n/a	-0.40
A. F Hernandez	NCT02465515	HARMONY	Albiglutide	placebo	8.70	8.76	-0.83	8.72	-0.31	-0.52
M.Pinget	NCT00763815	GETGOAL-P	Lixisenatide	placebo	8.07	8.08	-0.90	8.06	-0.34	-0.56
L. Ji	NCT03061214	SUSTAIN CHINA	Semaglutide	Sitagliptin	8.10	8.10	-1.60	8.10	-0.90	-0.70
R. Pratley	NCT00700817	LIRA-DPP-4	Liraglutide	Sitagliptin	8.40	8.40	-1.40	8.50	-0.88	-0.52
M. Pfeffer	NCT01147250	ELIXA	Lixisenatide	placebo	7.68	7.70	n/a	7.60	n/a	-0.27
C. Son		CANTABILE	Teneligliptin	Canaflozin	7.80	7.80	-0.65	7.70	-0.67	0.02
H. Rodbard	NCT02863328	PIONEER 2	Semaglutide	Empagliflozin	8.10	8.10	-1.30	8.10	-0.90	-0.40
B. Zinman	NCT01131676	EMPA-REG OUTCOME	Empagliflozin	placebo	8.08	8.07	n/a	8.08	n/a	-0.30
V. Perkovic	NCT02065791	CREDENCE	Canaflozin	placebo	8.30	8.30	-0.43	8.30	-0.32	-0.11
C. Cannon	NCT01986881	VERTIS CV	Ertugliflozin	placebo	8.20	8.20	-0.20	8.20	0.14	-0.34
S. Wiviott	NCT01730534	DECLARE-TIMI58	Dapagliflozin	placebo	8.30	8.30	-0.40	8.30	-0.20	-0.20
J. Rosenstock	NCT01897532	CARMELINA	Linagliptin	placebo	7.95	7.90	n/a	8.00	n/a	-0.36

B. Neal	NCT01032629	CANVAS	Canaflozin	placebo	8.30	8.30	-0.29	8.30	0.01	-0.30
G.Ledesma	NCT02240680		Linagliptin	placebo	8.20	8.20	-1.01	8.10	-0.38	-0.63
S. Marso	NCT01720446	SUSTAIN 6	Semaglutide	placebo	8.70	8.70	-1.25	8.70	-0.40	-0.85
A.Barnett	NCT00757588		Saxagliptin	placebo	8.65	12.20	-0.73	8.60	-0.32	-0.41
W. White	NCT00968708	EXAMINE	Alogliptin	placebo	8.00	8.00	-0.33	8.00	0.03	-0.36
C. Kovacs	NCT01210001	EMPA-REG EXTEND PIO	Empagliflozin	placebo	8.09	8.00	-0.65	8.16	-0.11	-0.54
J. Dou	NCT02273050	START	Saxagliptin	placebo	9.45	9.40	-3.00	9.50	-2.79	-0.21
H. Yki-Järvinen	NCT00954447		Linagliptin	placebo	8.30	8.31	-0.48	8.29	0.05	-0.53
Y. Chen	NCT02104804	SUPER	Saxagliptin	placebo	8.53	8.52	-0.64	8.53	-0.06	-0.58
J.Frias	NCT03353350	AMPLITUDE-M	Efpeglenatide	placebo	8.05	8.10	-1.40	8.00	-0.52	-0.88
	NCT00849056		Albiglutide	placebo	8.11	n/a	-0.81	n/a	-0.05	-0.76
	NCT01098539		Albiglutide	Sitagliptin	8.18	8.13	-1.04	8.23	-1.03	-0.01
B.Neal	NCT01989754	CANVAS-R	Canaflozin	placebo	8.30	n/a	n/a	n/a	n/a	-0.58
B. Ahren	NCT00838903	HARMONY 3	Albiglutide	Sitagliptin	8.13	8.10	-0.63	8.10	-0.28	-0.35
B. Ahren (2)	NCT00838903	HARMONY 3	Albiglutide	placebo	8.13	8.10	-0.63	8.20	0.27	-0.90
B. Ahren (3)	NCT00838903	HARMONY 3	Sitagliptin	placebo	8.13	8.10	-0.28	8.20	0.27	-0.55
	NCT00839527		Albiglutide	placebo	8.20	8.20	-0.53	8.30	0.33	-0.86
D. Bhatt	NCT03315143	SCORED	Sotagliflozin	placebo	8.30	8.30	-0.60	8.30	-0.17	-0.43
M. Riddle	NCT00715624	GETGOAL-L	Lixisenatide	placebo	8.40	8.42	-0.74	8.37	-0.38	-0.36
B. Scirica	NCT01107886	SAVOR- TIMI 53	Saxagliptin	placebo	8.00	8.00	-0.30	8.00	-0.10	-0.20
Hertzel C. Gerstein	NCT03496298	AMPLITUDE-O	Efpeglenatide	placebo	8.91	8.90	-1.42	8.94	-0.17	-1.25

Y. Seino	NCT00866658	GETGOAL-L-ASIA	Lixisenatide	placebo	8.53	8.54	-0.77	8.52	0.11	-0.88
I.Gantz	NCT01703208		Omargliptin	placebo	8.01	8.00	-0.36	8.00	-0.06	-0.30
J.Green	NCT00790205	TECOS	Sitagliptin	placebo	7.20	7.20	-0.10	7.20	0.10	-0.20
J.Rosenstock	NCT00713830	GETGOAL-S	Lixisenatide	placebo	8.25	8.10	-0.90	8.20	-0.10	-0.80
D. R. Owens	NCT00602472		Linagliptin	placebo	8.14	8.15	-0.72	8.14	-0.10	-0.62
B.Ahren	NCT00712673	GETGOAL-M	Lixisenatide	placebo	8.06	8.05	-0.81	8.10	-0.38	-0.43
R. Holman	NCT01144338	EXSCEL	Exenatide	placebo	8.00	8.00	n/a	8.00	n/a	-0.53
D.Matthews	NCT01528254	VERIFY	Vildagliptin	placebo	6.70	6.70	n/a	6.70	n/a	n/a
M.Sugawara		J-SELECT	Luseogliflozin	DPP-4i	7.65	7.7	-0.7	7.60	-0.60	-0.1
M.Davies	NCT03552757	STEP 2	Semaglutide	placebo	8.1	8.1	-1.6	8.10	-0.30	-1.3
B. Zinman	NCT03021187	PIONEER 8	Semaglutide	placebo	8.2	8.2	-0.9	8.20	-0.20	-0.7
V. Aroda	NCT02906930	PIONEER 1	Semaglutide	placebo	8	8	-1.23	7.90	-0.30	-0.93
Y. Seino	NCT02254291		Semaglutide	Sitagliptin	8.1	8.1	-2.05	8.20	-0.70	-1.35
D. Russell-Jones	NCT00331851	LEAD - 5 met+SU	Liraglutide	placebo	8.3	8.3	-1.33	8.30	-0.24	-1.09
Y. Seino	NCT01572740		Liraglutide	placebo	8.8	8.8	-1.68	8.80	-0.88	-0.8
W. Wang	NCT04591626	AWARD-CHN3	Dulaglutide	placebo	8.6	8.6	-2.03	8.60	-1.08	-0.95
	NCT04017832	PIONEER 12	Semaglutide	Sitagliptin			-1.23		-0.70	-0.53
W.Yang	NCT01095666		Dapagliflozin	placebo	8.13	8.13	-0.835	8.13	-0.23	-0.6
Y. Seino	NCT00395746		Liraglutide	placebo	8.82	8.805	-1.185	8.85	-0.05	-1.18
J. Rosenstock	NCT01011868	EMPA-REG BASAL	Empagliflozin	placebo	8.2	8.3	-0.65	8.20	0.00	-0.65
Y. Yamada	NCT03018028	PIONEER 9	Semaglutide	placebo	8.2	8.2	-1.3	8.30	0.10	-1.4
T.Pieber	NCT02849080	PIONEER 7	Semaglutide	Sitagliptin	8.3	8.3	-1.3	8.30	-0.80	-0.5
O.Mosenzon	NCT02827708	PIONEER 5	Semaglutide	Placebo	8	8	-1.1	7.90	-0.20	-0.9

H. Rodbard	NCT02305381	SUSTAIN 5	Semaglutide	Placebo	8.4	8.35	-1.6	8.4	-0.1	-1.5

CTID, ClinicalTrials.gov identifier; IG, intervention group; CG, control group; n/a, non-available;

Table S2. Risk of bias assessment.

**Study**	**D1**	**D2**	**D3**	**D4**	**D5**	**OA**
Husain (2019)	Green	Green	Green	Green	Green	Green
Rosenstock (2019)	Green	Yellow	Green	Green	Green	Yellow
Zinman (2019)	Green	Green	Green	Green	Green	Green
Gerstein (2019)	Green	Green	Green	Yellow	Green	Yellow
Nauck (2016)	Green	Yellow	Green	Green	Green	Yellow
Marso (2016)	Green	Green	Green	Yellow	Green	Yellow
Hernandez (2018)	Green	Yellow	Green	Yellow	Green	Yellow
Pinget (2013)	Green	Green	Red	Yellow	Green	Red
Ji (2021)	Green	Yellow	Green	Green	Green	Yellow
Pratley (2010)	Red	Green	Green	Red	Green	Red
Pfeffer (2015)	Green	Green	Green	Red	Green	Red
Son (2021)	Red	Yellow	Green	Red	Green	Red
Rodbard (2019)	Red	Yellow	Green	Green	Green	Red
Zinman (2015)	Green	Green	Green	Red	Green	Red
Perkovic (2019)	Green	Yellow	Green	Yellow	Green	Yellow
Cannon (2020)	Green	Green	Green	Red	Green	Red
Wiviott (2019)	Green	Green	Green	Yellow	Yellow	Yellow
Rosenstock (2019)	Green	Green	Green	Yellow	Green	Yellow
Neal (2017)	Green	Green	Green	Yellow	Green	Yellow
Ledesma (2019)	Green	Green	Green	Red	Green	Red
Marso (2016)	Green	Green	Green	Green	Green	Green
Barnett (2012)	Green	Green	Green	Yellow	Green	Yellow
White (2013)	Green	Green	Green	Yellow	Green	Yellow
Kovacs (2015)	Green	Green	Green	Yellow	Green	Yellow
Dou (2018)	Green	Yellow	Green	Yellow	Green	Yellow
Yki-Jarvinen (2013)	Green	Green	Green	Yellow	Green	Yellow

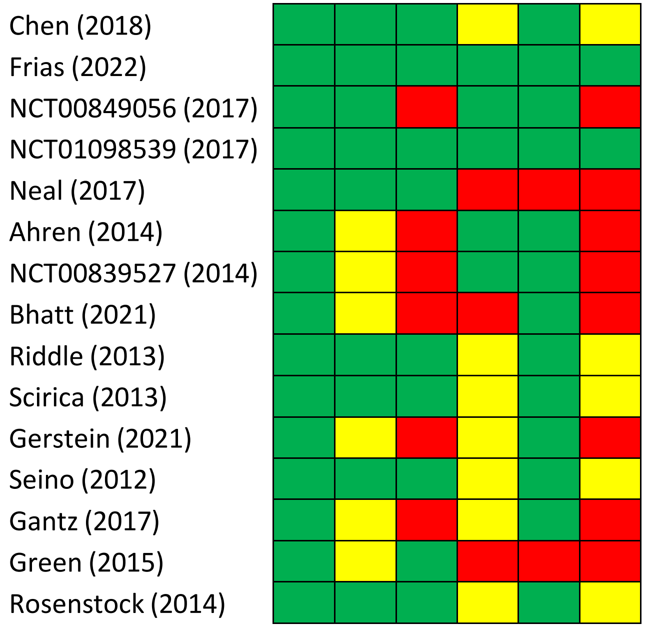
Chen (2018)
Frias (2022)
NCT00849056 (2017)
NCT01098539 (2017)
Neal (2017)
Ahren (2014)
NCT00839527 (2014)
Bhatt (2021)
Riddle (2013)
Scirica (2013)
Gerstein (2021)
Seino (2012)
Gantz (2017)
Green (2015)
Rosenstock (2014)

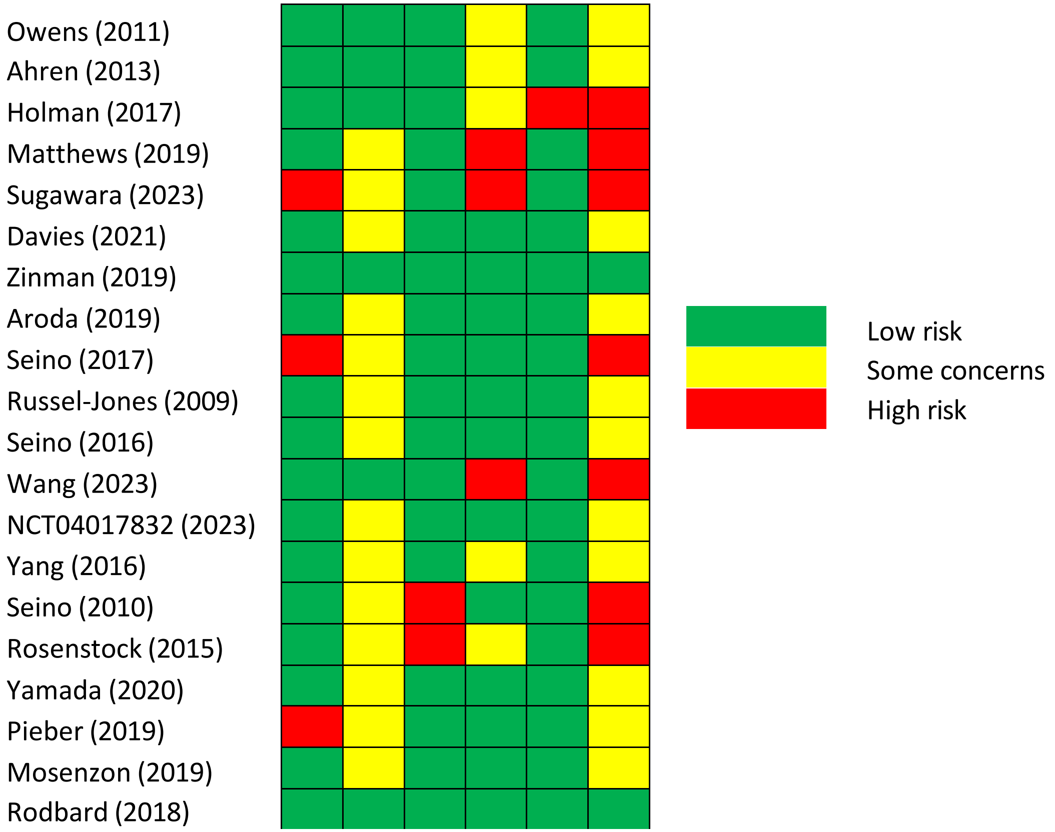

D1, Randomization process; D2, Deviations from the intended interventions; D3, Missing outcome data; D4, Measurement of the outcome; D5, Selection of the reported result; OA, overall;

Table S3. Certainty assessment.

Outcome	Number of studies	Risk of bias	Inconsistency	Indirectness	Imprecision	Publication bias	Overall	Absolute risk
GLP-1RA vs Placebo	29	Not serious	Not serious	Serious	Not serious	Not serious	Moderate	2 more per 1000 (from 1 fewer to 5 more)
DPP-4i vs Placebo	13	Not serious	Not serious	Serious	Not serious	Not serious	Moderate	1 more per 1000 (from 1 fewer to 4 more)
SGLT-2 vs Placebo	10	Serious	Not serious	Serious	Not serious	Not serious	Low	0 more per 1000 (from 1 fewer to 2 more)
GLP-1RA vs DPP-4i	8	Not serious	Serious	Serious	Serious	Not serious	Very low	7 fewer per 1000 (from 28 fewer to 30 more)
Canagliflozin vs Placebo	3	Not serious	Not serious	Serious	Not serious	Not serious	Moderate	4 more per 1000 (from 1 fewer to 9 more)
Empagliflozin vs Placebo	3	Serious	Not serious	Serious	Not serious	Not serious	Low	3 fewer per 1000 (from 4 fewer to 1 fewer)
Linagliptin vs Placebo	4	Not serious	Not serious	Serious	Not serious	Serious	Low	3 fewer per 1000 (from 5 fewer to 2 more)
Saxagliptin vs Placebo	4	Not serious	Not serious	Serious	Not serious	Not serious	Moderate	1 more per 1000 (from 0 fewer to 3 more)
Albiglutide vs Placebo	5	Serious	Not serious	Serious	Serious	Serious	Very low	3 more per 1000 (from 5 fewer to 14 more)
Lixisenatide vs Placebo	6	Not serious	Not serious	Serious	Not serious	Not serious	Moderate	0 fewer per 1000 (from 1 fewer to 2 more)
Semaglutide vs Placebo	9	Not serious	Not serious	Serious	Serious	Not serious	Very low	6 more per 1000 (from 5 fewer to 22 more)
Liraglutide vs Placebo	4	Not serious	Not serious	Serious	Serious	Serious	Very low	1 more per 1000 (from 5 fewer to 10 more)

Figure S2: Pairwise meta-analysis, GLP-1RA vs DPP-4i [21,28,29,47,64,72].

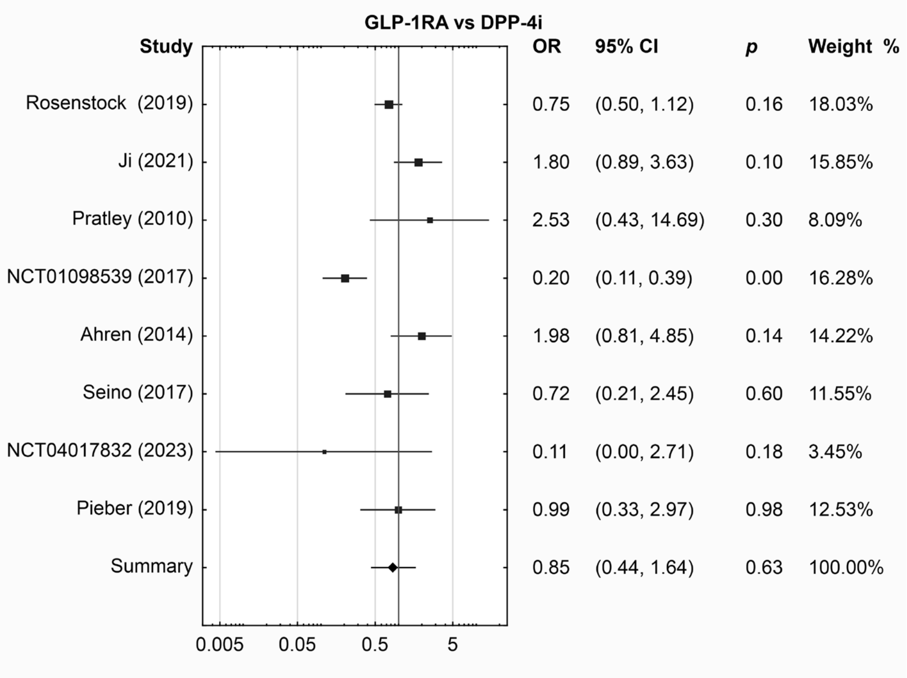

Figure S3. Pairwise meta-analysis, Semaglutide vs placebo [20,22,6,61,62,63,71,73,74].

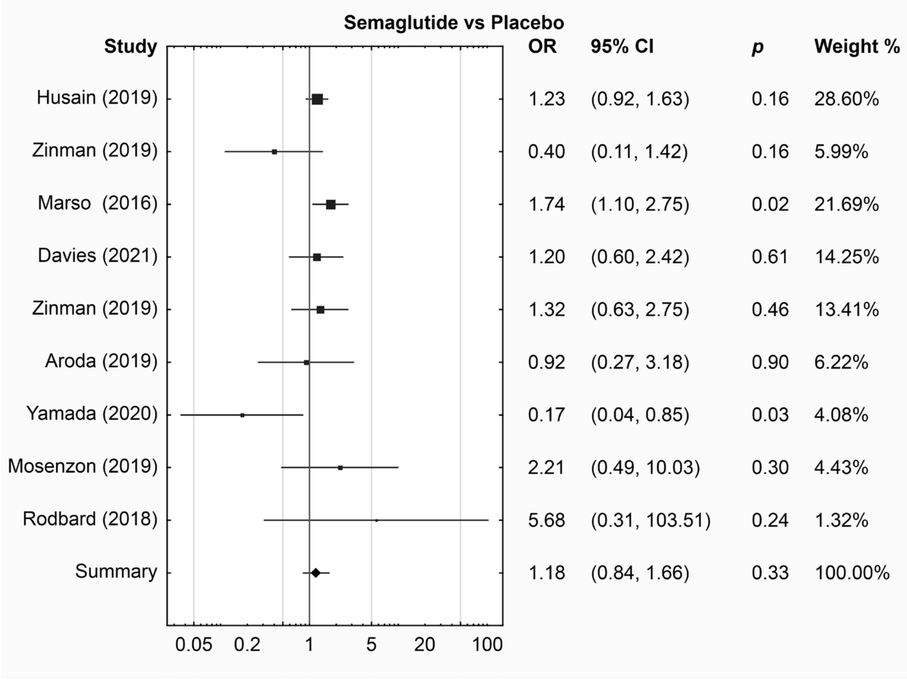

Figure S4. Pairwise meta-analysis, Liraglutide vs placebo [25,65,66,69].

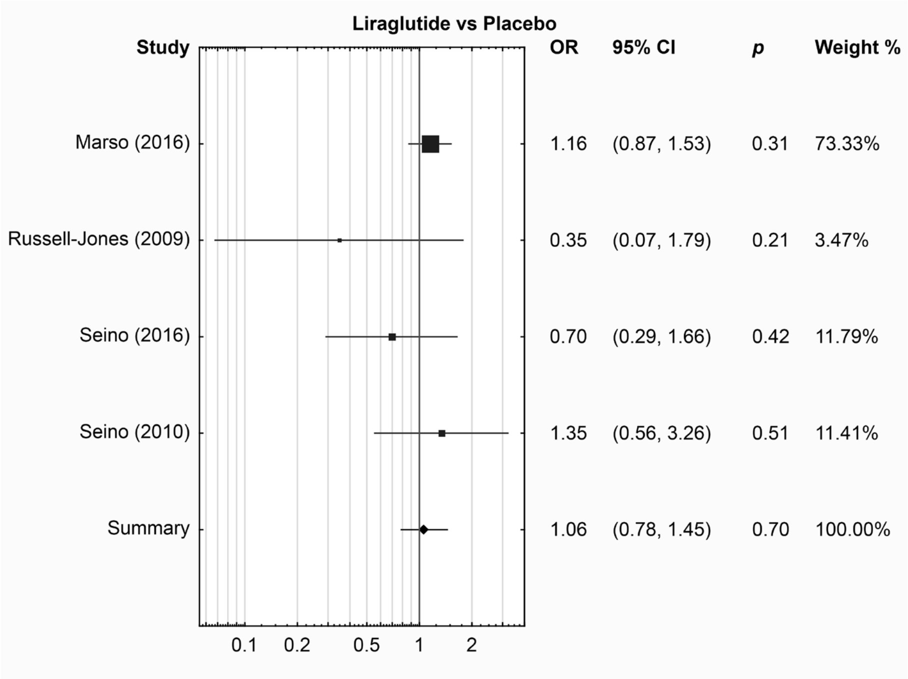

Figure S5. Pairwise meta-analysis, Albiglutide vs placebo [24,26,47].

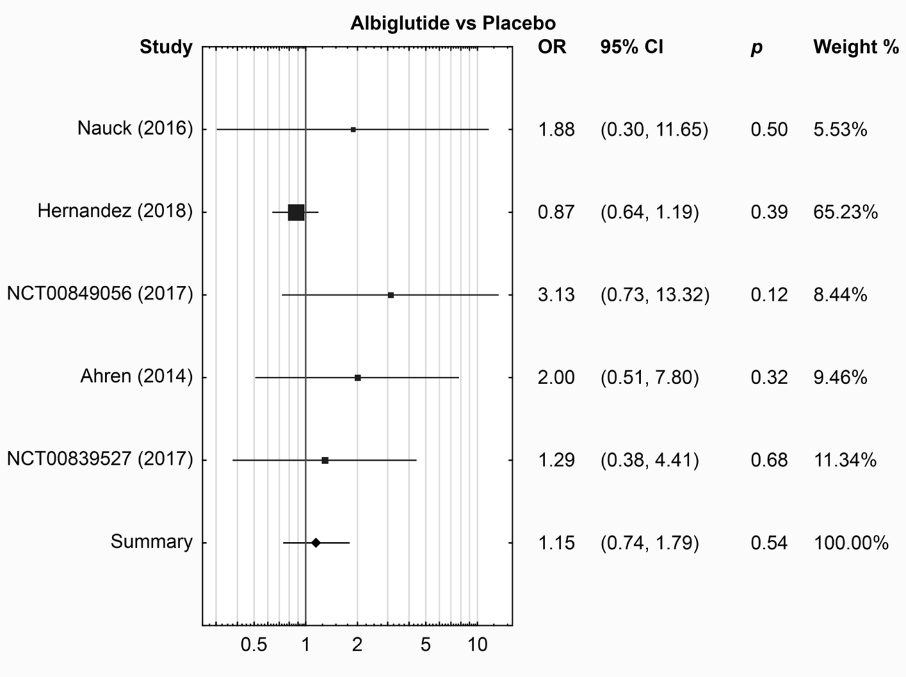

Figure S6. Pairwise meta-analysis, Lixisenatide vs placebo [27,30,49,52,55,57].

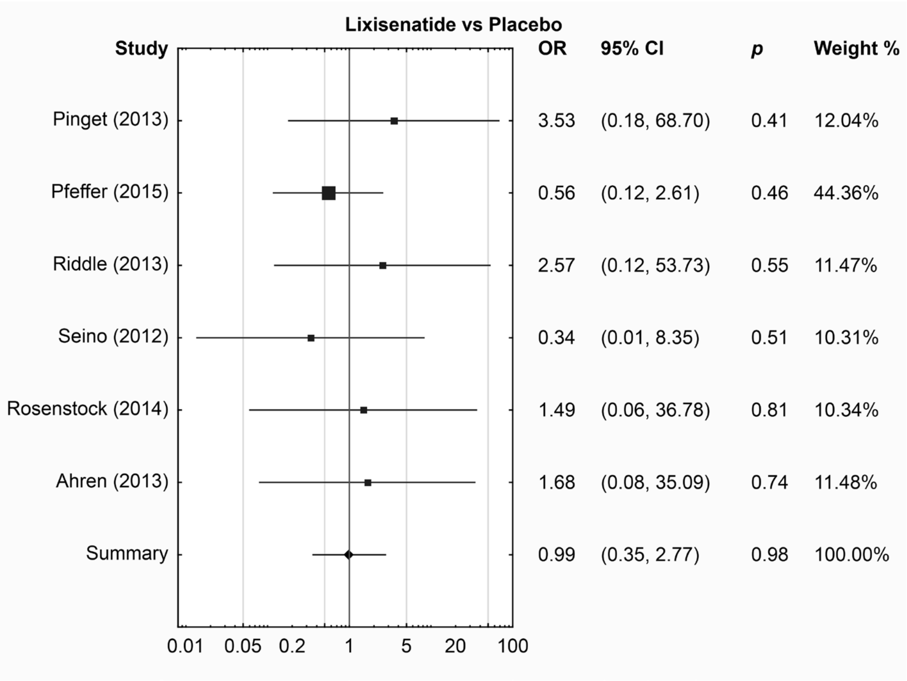

Figure S7. Pairwise meta-analysis, Linagliptin vs placebo [37,39,44,56].

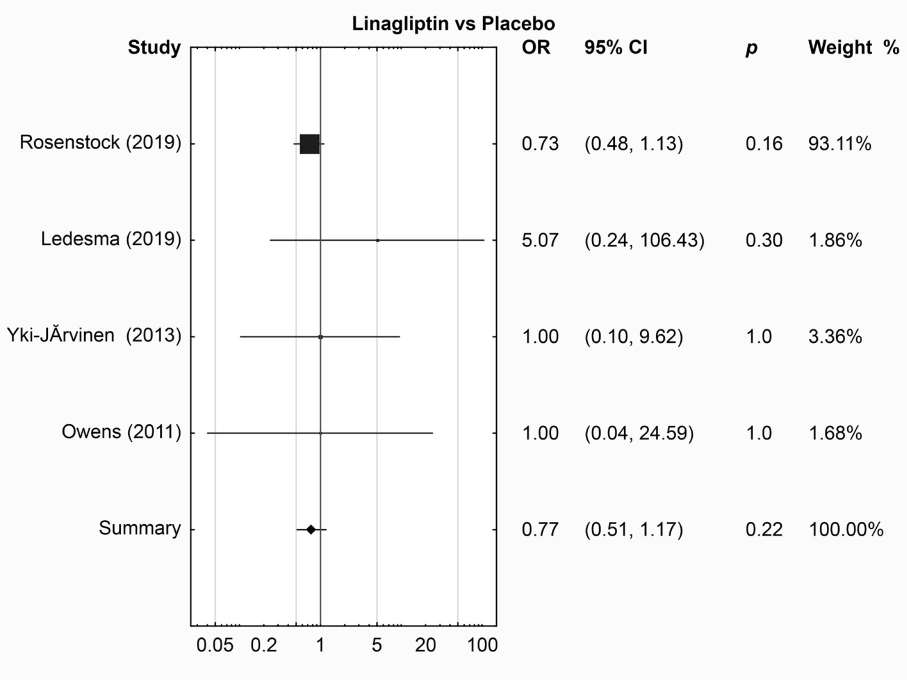

Figure S8. Pairwise meta-analysis, Saxagliptin vs placebo [40, 43,45,50].

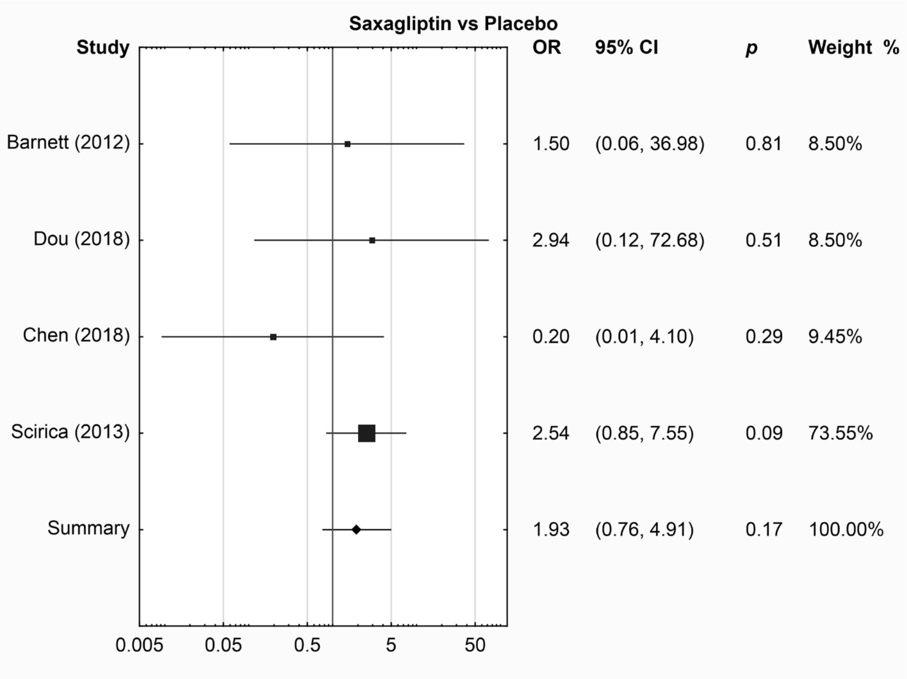

Figure S9. Pairwise meta-analysis, Canagliflozin vs placebo [34,38].

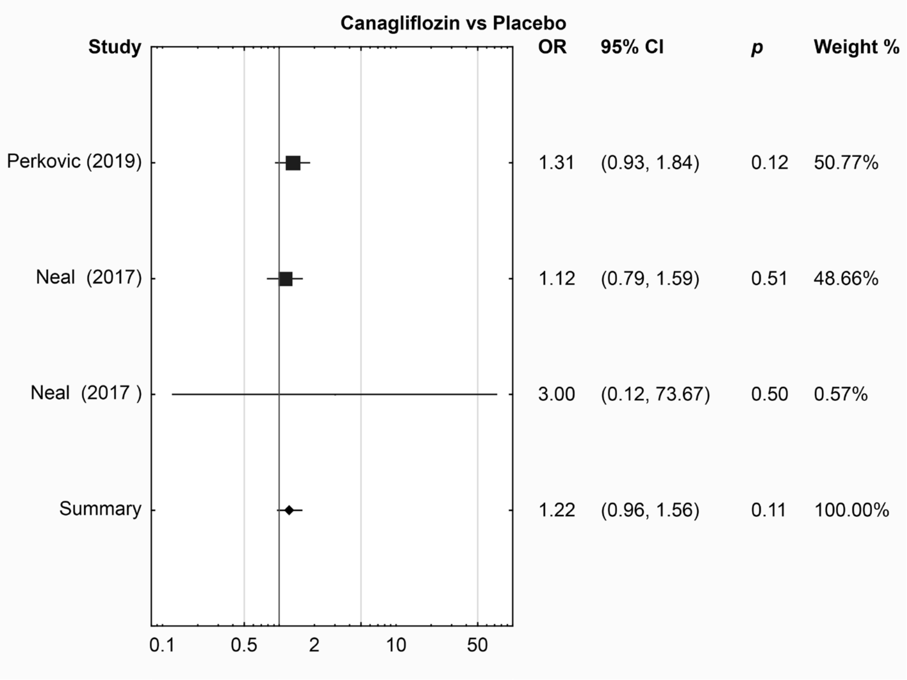

Figure S10: Pairwise meta-analysis, Empagliflozin vs placebo [33,42,70].

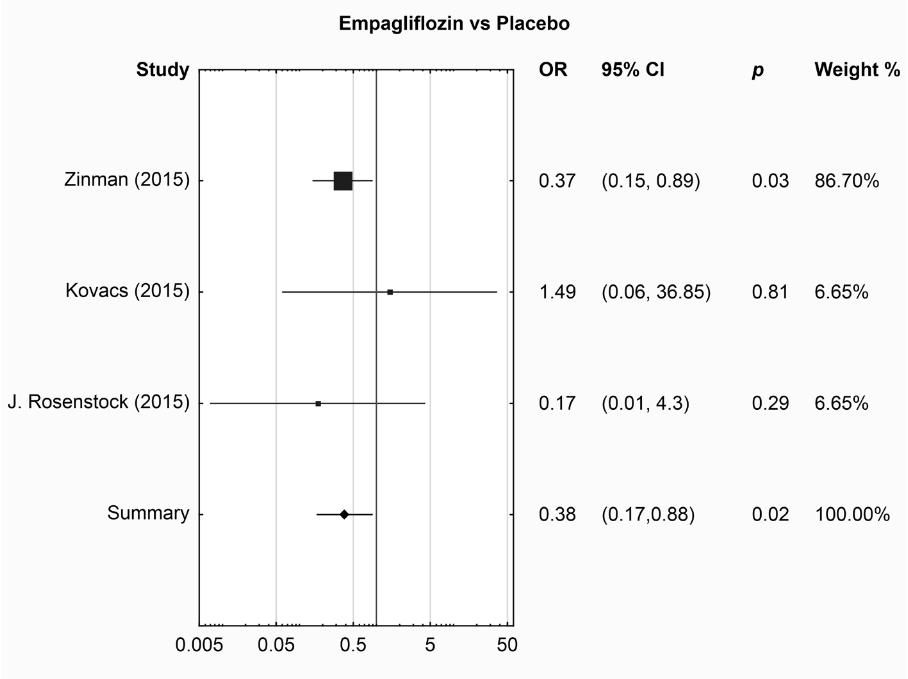

Table S4. Meta-regression of prespecified trails characteristics on odds ratio of diabetic retinopathy incidents. Multivariate model. Log OR, 44 trials. random-effects model. R2= 0.00%. I2= 11.24%. Q residual = 42.35, Q combined = 48.45.

Factor	b	Standard Error	95% CI LL	95% CI UL	Z	p
Intercept	-2.31	2.27	-6.76	2.13	-1.02	0.3082
Age	0.01	0.02	-0.03	0.06	0.59	0.5566
Diabetes duration	-0.02	0.03	-0.08	0.05	-0.49	0.6264
BMI at baseline	0.06	0.03	0.00	0.13	1.87	0.0620
HbA1C at baseline	-0.03	0.15	-0.32	0.25	-0.21	0.8307
HbA1C change intervention - control	0.15	0.20	-0.25	0.55	0.74	0.4574

CI, confidence interval; LL, lower limit; UL, upper limit;

Table S5. Meta-regression of prespecified trails characteristics on odds ratio of diabetic retinopathy incidents. Univariate model. GLP-1RA vs Placebo. Log OR, 29 trials, random-effects model. R2= 78.32%. I2= 13.82%. Q residual = 27.95, Q combined 32.49.

Factor	b	Standard Error	95% CI LL	95% CI UL	Z	p
Intercept	-2.41	1.19	-4.75	-0.07	-2.02	0.0436
BMI	0.08	0.04	0.00	0.15	2.07	0.0383

CI, confidence interval; LL, lower limit; UL, upper limit;

Table S6. Meta-regression of prespecified trails characteristics on odds ratio of diabetic retinopathy incidents. Univariate model. DPP-4i vs Placebo**.** Log OR, 12 trials, random-effects model. R2 = 100.00%. I2 = 13.22%. Q residual = 8.61, Q combined = 12.68.

Factor	b	Standard Error	95% CI LL	95% CI UL	Z	p
Intercept	0.65	0.27	0.13	1.18	2.44	0.0148
HbA1C change intervention - control	2.14	1.06	0.06	4.21	2.02	0.0437

CI, confidence interval; LL, lower limit; UL, upper limit;

Table S7. Meta-regression of prespecified trails characteristics on odds ratio of diabetic retinopathy

incidents. Univariate model. SGLT-2i vs Placebo. Log OR, 10 trials, random-effects model. R2= 100%. I2= 19.34%. Q residual = 3.43, Q combined = 11.16.

**Factor**	**b**	**Standard Error**	**95% CI LL**	**95% CI UL**	**Z**	**p**
Intercept	-43.84	15.80	-74.81	-12.88	-2.78	0.0055
HbA1C baseline	5.30	1.91	1.57	9.04	2.78	0.0054

CI, confidence interval; LL, lower limit; UL, upper limit;

Table S8 Meta-regression of prespecified trails characteristics on odds ratio of diabetic retinopathy incidents. Univariate model. GLP-1RA vs DPP-4i. Log OR, 8 trials, random-effects model. R2= 100%. I2= 76.16%. Q residual = 4.41, Q combined 29.36.

**Factor**	**b**	**Standard Error**	**95% CI LL**	**95% CI UL**	**Z**	**p**
Intercept	12.28	2.52	7.35	17.22	4.88	0.0000
Age	-0.22	0.04	-0.30	-0.13	-5.00	0.0000

CI, confidence interval; LL, lower limit; UL, upper limit;

Table S9 Meta-regression of prespecified trails characteristics on odds ratio of diabetic retinopathy incidents. Univariate model. GLP-1RA vs DPP-4i. Log OR, 7 trials, random-effects model. R2= 100%. I2= 78.53%. Q residual = 0.94, Q combined 27.95.

**Factor**	**b**	**Standard Error**	**95% CI LL**	**95% CI UL**	**Z**	**p**
Intercept	3.47	0.73	2.04	4.90	4.76	0.0000
Diabetes duration	-0.44	0.08	-0.61	-0.28	-5.20	0.0000

CI, confidence interval; LL, lower limit; UL, upper limit;

Figure S11. Prisma checklist. Part 1 [104].

Section and Topic	Item #	Checklist item	Reported on page:
**TITLE**			
Title	1	Identify the report as a systematic review.	1
**ABSTRACT**			
Abstract	2	See the PRISMA 2020 for Abstracts checklist.	1
**INTRODUCTION**			
Rationale	3	Describe the rationale for the review in the context of existing knowledge.	1-2
Objectives	4	Provide an explicit statement of the objective(s) or question(s) the review addresses.	2
**METHODS**			
Eligibility criteria	5	Specify the inclusion and exclusion criteria for the review and how studies were grouped for the syntheses.	2
Information sources	6	Specify all databases, registers, websites, organisations, reference lists and other sources searched or consulted to identify studies. Specify the date when each source was last searched or consulted.	2
Search strategy	7	Present the full search strategies for all databases, registers and websites, including any filters and limits used.	Supplement p 1
Selection process	8	Specify the methods used to decide whether a study met the inclusion criteria of the review, including how many reviewers screened each record and each report retrieved, whether they worked independently, and if applicable, details of automation tools used in the process.	2
Data collection process	9	Specify the methods used to collect data from reports, including how many reviewers collected data from each report, whether they worked independently, any processes for obtaining or confirming data from study investigators, and if applicable, details of automation tools used in the process.	2
Data items	10a	List and define all outcomes for which data were sought. Specify whether all results that were compatible with each outcome domain in each study were sought (e.g. for all measures, time points, analyses), and if not, the methods used to decide which results to collect.	2-3
	10b	List and define all other variables for which data were sought (e.g. participant and intervention characteristics, funding sources). Describe any assumptions made about any missing or unclear information.	2
Study risk of bias assessment	11	Specify the methods used to assess risk of bias in the included studies, including details of the tool(s) used, how many reviewers assessed each study and whether they worked independently, and if applicable, details of automation tools used in the process.	2
Effect measures	12	Specify for each outcome the effect measure(s) (e.g. risk ratio, mean difference) used in the synthesis or presentation of results.	3
Synthesis methods	13a	Describe the processes used to decide which studies were eligible for each synthesis (e.g. tabulating the study intervention characteristics and comparing against the planned groups for each synthesis).	3
	13b	Describe any methods required to prepare the data for presentation or synthesis, such as handling of missing summary statistics, or data conversions.	2-3
	13c	Describe any methods used to tabulate or visually display results of individual studies and syntheses.	3
	13d	Describe any methods used to synthesize results and provide a rationale for the choice(s). If meta-analysis was performed, describe the model(s), method(s) to identify the presence and extent of statistical heterogeneity, and software package(s) used.	3
	13e	Describe any methods used to explore possible causes of heterogeneity among study results (e.g. subgroup analysis, meta-regression).	3
	13f	Describe any sensitivity analyses conducted to assess robustness of the synthesized results.	3
Reporting bias assessment	14	Describe any methods used to assess risk of bias due to missing results in a synthesis (arising from reporting biases).	2
Certainty assessment	15	Describe any methods used to assess certainty (or confidence) in the body of evidence for an outcome.	3

Figure S12. Prisma checklist. Part 2 [104].

Section and Topic	Item #	Checklist item	Location where item is reported
**RESULTS**			
Study selection	16a	Describe the results of the search and selection process, from the number of records identified in the search to the number of studies included in the review, ideally using a flow diagram.	Supplement p 14
	16b	Cite studies that might appear to meet the inclusion criteria, but which were excluded, and explain why they were excluded.	n/a
Study characteristics	17	Cite each included study and present its characteristics.	3-7
Risk of bias in studies	18	Present assessments of risk of bias for each included study.	Supplement p 7-8
Results of individual studies	19	For all outcomes, present, for each study: (a) summary statistics for each group (where appropriate) and (b) an effect estimate and its precision (e.g. confidence/credible interval), ideally using structured tables or plots.	9-1, Supplement 11-14
Results of syntheses	20a	For each synthesis, briefly summarise the characteristics and risk of bias among contributing studies.	8, Supplement p 6
	20b	Present results of all statistical syntheses conducted. If meta-analysis was done, present for each the summary estimate and its precision (e.g. confidence/credible interval) and measures of statistical heterogeneity. If comparing groups, describe the direction of the effect.	8
	20c	Present results of all investigations of possible causes of heterogeneity among study results.	11
	20d	Present results of all sensitivity analyses conducted to assess the robustness of the synthesized results.	12
Reporting biases	21	Present assessments of risk of bias due to missing results (arising from reporting biases) for each synthesis assessed.	11
Certainty of evidence	22	Present assessments of certainty (or confidence) in the body of evidence for each outcome assessed.	7-8, Supplement p 6
**DISCUSSION**			
Discussion	23a	Provide a general interpretation of the results in the context of other evidence.	12-15
	23b	Discuss any limitations of the evidence included in the review.	14-15
	23c	Discuss any limitations of the review processes used.	14-15
	23d	Discuss implications of the results for practice, policy, and future research.	15
**OTHER INFORMATION**			
Registration and protocol	24a	Provide registration information for the review, including register name and registration number, or state that the review was not registered.	2
	24b	Indicate where the review protocol can be accessed, or state that a protocol was not prepared.	2
	24c	Describe and explain any amendments to information provided at registration or in the protocol.	n/a
Support	25	Describe sources of financial or non-financial support for the review, and the role of the funders or sponsors in the review.	16
Competing interests	26	Declare any competing interests of review authors.	16
Availability of data, code and other materials	27	Report which of the following are publicly available and where they can be found: template data collection forms; data extracted from included studies; data used for all analyses; analytic code; any other materials used in the review.	n/a
